# Salivary glycopatterns as potential biomarkers for diagnosis of gastric cancer

**DOI:** 10.18632/oncotarget.16082

**Published:** 2017-03-10

**Authors:** Jian Shu, Hanjie Yu, Xiaojie Li, Dandan Zhang, Xiawei Liu, Haoqi Du, Jiaxu Zhang, Zhao Yang, Hailong Xie, Zheng Li

**Affiliations:** ^1^ Laboratory for Functional Glycomics, College of Life Sciences, Northwest University, Xi'an, China; ^2^ Department of Pothology. First People`s Hospital of Chenzhou, Chenzhou, China; ^3^ Institute of Cancer Research, University of South China, Hengyang, China

**Keywords:** salivary glycopatterns, gastric cancer, biomarker, diagnostic models

## Abstract

Gastric cancer (GC) is still an extremely severe health issue with high mortality due to the lacking of effective biomarkers. In this study, we aimed to investigate the alterations of salivary protein glycosylation related to GC and assess the possibility of salivary glycopatterns as potential biomarkers for the diagnosis of GC. Firstly, 94 patients with GC (*n* = 64) and atrophic gastritis (AG) (*n* = 30), as well as 30 age- and sex-matched healthy volunteers (HV) were enrolled in the test group to probe the difference of salivary glycopatterns using lectin microarrays, the results were validated by saliva microarrays and lectin blotting analysis. Then, the diagnostic model of GC (Model GC) and AG (Model AG) were constructed based on 15 candidate lectins which exhibited significant alterations of salivary glycopattern by logistic stepwise regression. Finally, two diagnostic models were assessed in the validation group including HV (*n* = 30) and patients with GC (*n* = 23) and AG (*n* = 24) and achieved high diagnostic power (Model GC (AUC: 0.89, sensitivity: 0.96 and specificity: 0.80), Model AG (AUC: 0.83, sensitivity: 0.92 and specificity: 0.72)). This study provides pivotal information to distinguish HV, AG and GC based on precise alterations in salivary glycopatterns, which have great potential to be biomarkers for diagnosis of GC.

## INTRODUCTION

Gastric cancer (GC) is a kind of malignant tumor with high incidence and mortality especially in developing countries. It affects approximately one million individuals per year worldwide [1]. Pathological analyses demonstrated that most GC cases are closely associated with gastritis, and most gastritis cases experience a series of sequential gradual evolution steps in the coming years and decades, including acute gastritis, chronic atrophic gastritis, intestinal metaplasia, dysplasia, and adenocarcinoma [2, 3]. Previous researchers have discovered several glycoprotein biomarkers (CA72-4, CA19-9, CEA, CA125) [4, 5]. Unfortunately, all of the existing biomarkers are not enough sensitive or specific to characterize the early GC [6]. About 80% patients were diagnosed only at the advanced stages which makes high mortality [7]. Therefore, discovering effective biomarkers for accurately distinguishing early GC is an urgent assignment.

The glycosylation of proteins and lipids are arguably the most abundant posttranslational modifications. Recent advances in glycomics reveal the scope and scale of their functional roles and their impact on human disease, which has become an emerging hot research area in cancer-biology with the contribution of molecular mechanism research as well as clinical auxiliary diagnosis [8, 9]. Glycans occurs on cell surface membrane-anchored and the secreted glycoproteins creating the initial site of contact in cellular and extracellular interactions, which closely reflects the physiological status of the cells [10, 11]. Therefore, exploring the correlation between glycans and disease is more evident than that of cancer related changes in proteins. Many evidences showed that glycosylation is directly associated with tumor cell development. In the process of gastric malignant transformation, E-cadherin and integrin have been glycosylated by N-acetylglucosaminyltransferase-V, leading to a rapid up-regulation in β1, 6-GlcNAc branched N-glycan [9, 12]. It reduces cell-cell, cell-extracellular matrix adhesion and contributes to tumor cell invasion and metastases [9].

Saliva is a good indicator of the plasma levels of various substances, which is a mirror of body health [13]. Salivary proteins has been extensively used for disease diagnosis in different fields [14–16]. Recent studies have also elucidated that the salivary proteins could also be used for the non-invasive detection of gastric cancer [17]. And glycosylation alterations of human salivary glycoproteins frequently occurred during several disease and cancer progression [18]. Our previous study revealed that the sex/age-associated differences in the glycopatterns of healthy human salivary glycoproteins. Healthy elderly individuals are found to have stronger resistance to influenza A virus (IAV) partly by presenting more terminal α2-3/6-linked sialic acid residues in their saliva to inhibit the activities of IAV which provides the evidence that elderly individuals with chronic diseases, such as diabetes and liver disease, might be more susceptible to avian influenza viruses due to the decreased expression of terminal α2-3-linked sialic acids in their saliva [19, 20]. Lectins are carbohydrate-binding proteins that discriminate between glycopatterns of glycans based on subtle differences in structure. Several lectins, including VVA, PNA, PSA, LEL and SBA, are generally used to study altered glycans structures in gastric cancer [21, 22]. The advent of high-throughput glycomic techniques enabled the lectin microarrays to observe multiple, distinct binding interactions simultaneously, which have become a primary method to investigate glycosylation of crude samples [23, 24].

The purpose of this study is to investigate the correlation of alterations in salivary protein glycosylation related to GC and compare different or similar alterations of glycoprotein glycopatterns among healthy volunteers (HV), atrophic gastritis (AG), and GC (Adenocarcinoma of stage I/II/III) groups. Furthermore the possibility of salivary glycopatterns acting as potential biomarkers for diagnosis of GC was assessed.

## RESULTS

### Salivary glycopatterns in patients with gastric cancer or atrophic gastritis and healthy volunteers

**Figure 1:**
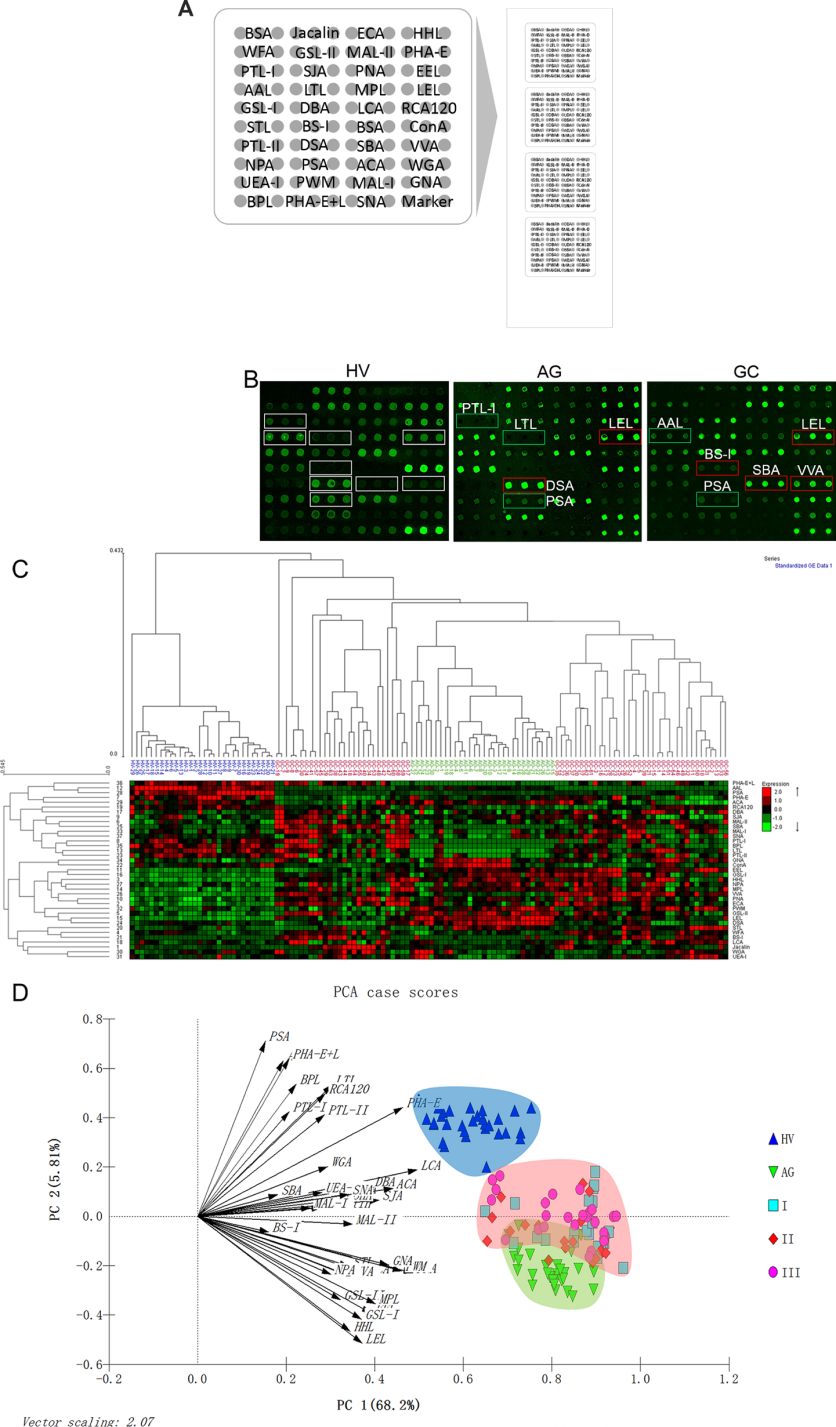
The different salivary glycopatterns in Patients with GC or AG and in HV using a lectin microarray (**A**) The layout of the lectin microarrays. Each lectin was spotted in triplicate per block, with quadruplicate blocks on one slide. Cy3-labeled BSA was spotted as a location marker and BSA as a negative control. (**B**) The glycopatterns of a Cy3-labeled salivary sample bound to the lectin microarrays. The lectin microarrays revealed significant increase marked with red frames while the significant decrease marked with green frames. (**C**) Unsupervised average linkage HCA of the lectin microarray responses to saliva. The samples were listed in columns, and the lectins were listed in rows. The color and intensity of each square indicated expression levels relative to the other data in the row. Red, high; green, low; black, medium. (**D**) The normalized glycopattern abundances responses to three pools were visualized by PCA. HV, AG and GC were indicated by a blue shadow, green shadow and red shadow, respectively.

All salivary samples included in the test group were tested using the lectin microarrays independently. The layout of the lectin microarrays and the Cy3-labeled salivary sample bound to the lectin microarrays were shown in Figure 1A and 1B. The generated data from each sample were executed by HCA using EXPANDER 6.0 to achieve the hierarchical relationship according to the similarities of the glycopatterns abundances. As shown in Figure 1C, 124 samples distributed in the heat map. The samples of HV and AG clustered closely, and GC achieved a closer hierarchical relationship with AG than HV, indicating that the expression levels of salivary glycopatterns were more similar between GC and AG. But there were no clear distinction among different stages of GC. The results indicated that salivary glycopatterns was possible to provide signature information as biomarker. Furthermore, PCA was performed to illustrate the relationships among the samples that placed as points in a 2-dimensional scatter plot for data visualization. The PCA results were generated based on the normalized fluorescent intensities (NFIs), which response the aggregate recognition power of each lectin for all salivary samples in the test group. The subjects assigned to scatterplots tended to cluster separately to form HV, AG, and GC pools with different colours and symbols in Figure 1D, which also indicated that it was possible to distinguish among HV, AG, and GC based on precise alterations in salivary glycopatterns. But as the same as HCA, it cannot distinguish different stages of GC. Interestingly, it showed that there were a small overlapping area between AG and GC pools, indicating that the salivary glycopattern expression levels of AG were partly similar to GC, which might imply an early stage of a malignant transformation from AG to GC.

### Alterations of salivary glycopatterns among healthy volunteers, gastric cancer, and atrophic gastritis patients

**Figure 2:**
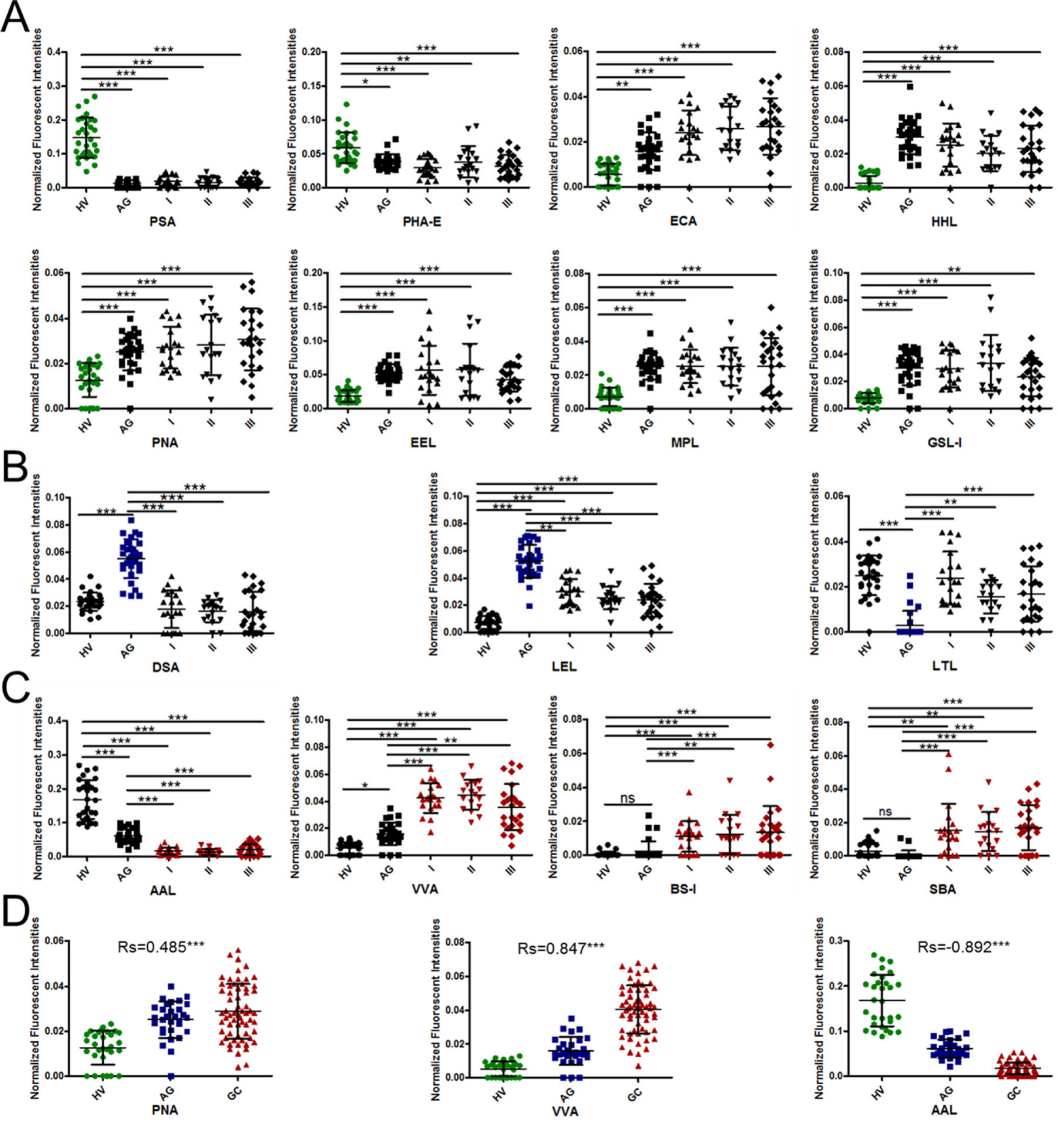
Alterations of salivary glycopatterns between patients with GC or AG and HV (**A**) The difference of salivary glycopatterns between HV and GC&AG. (**B**) The difference of salivary glycopatterns between AG and HV&GC. (**C**) The difference of salivary glycopatterns between GC and HV&AG. (**D**) The scatterplots of Spearman's correlation between pathological type and specific lectins. *P < 0.05; **P < 0.01; and ***P < 0.001.

The NFIs of each candidate lectin that showed variable expression levels of salivary glycopatterns were further represented in scatter diagram. Totally, there were 15 lectins that revealed significant alterations in salivary glycopatterns among HV, AG and GC (including different stages). As shown in Figure 2A, the Fucα-1,6GlcNAc (core fucose) binder PSA, and the bisecting GlcNAc and the biantennary complex-type N-glycan with outer Gal binder PHA-E exhibited significantly decreased NFI in all patients with AG or GC compared with the HV (all *p* < 0.05). However, the Galβ-1,4GlcNAc and Galβ1-3GlcNAc binder ECA, the high-Mannose, Manα1-3Man, and Manα1-6Man binder HHL, the Galβ1-3GalNAcα-Ser/Thr (T antigen) binder PNA, the Galα1-3(Fucα1-2)Gal (blood group B antigen) binder EEL, the T antigen, and GalNAc binder MPL, and the Galβ1-3GalNAc, αGalNAc, and αGal binder GSL-I exhibited significantly increased NFIs in all patients with AG or GC compared with the HV (all *p* < 0.01).

As shown in Figure 2B, the β1-4GlcNAc and LacNAc binder DSA, the (GlcNAc)n and high mannose-type N-glycan binder LEL exhibited significantly increased NFIs in AG compared with HV and GC (all *p* < 0.01), however, the Fucα1-2Galβ1-4GlcNAc and Fucα1-3(Galβ1-4)GlcNAc binder LTL exhibited significantly decreased NFIs in AG compared with HV and GC (all *p* < 0.01). As show in Figure 2C, the Fucα1-6 GlcNAc (core fucose) and Fucα1-3(Galβ1-4)GlcNAc binder AAL exhibited significantly decreased NFIs in GC compared with HV and AG (all *p* ≤ 0.001), however, the GalNAcα-Ser/Thr (Tn antigen)and GalNAc binder VVA, α-Gal, α-GalNAc, Galα-1,3Gal, and the Galα-1,6Glc binder BS-I, and the α- or β-linked terminal GalNAc, (GalNAc)n, and GalNAcα1-3Gal binder SBA exhibited significantly increased NFIs in GC compared with HV and AG (all *p* < 0.01).

The above results showed the significant alterations of salivary glycopatterns during the development of GC. However, there were no significant distinction among Stage I, Stage II, and Stage III of GC in salivary glycopatterns. In addition, the Spearman's correlation coefficients was performed to evaluate the correlations among HV, AG and GC in salivary glycopatterns. The scatterplots of Spearman's correlation for 124 samples from HV, AG and GC showed that the NFIs of PNA and VVA were positively correlated with the development of GC, and AAL were negatively correlated with the development of GC (Figure 2D).

### Validation of the gastric cancer-associated differences of salivary glycopatterns

**Figure 3:**
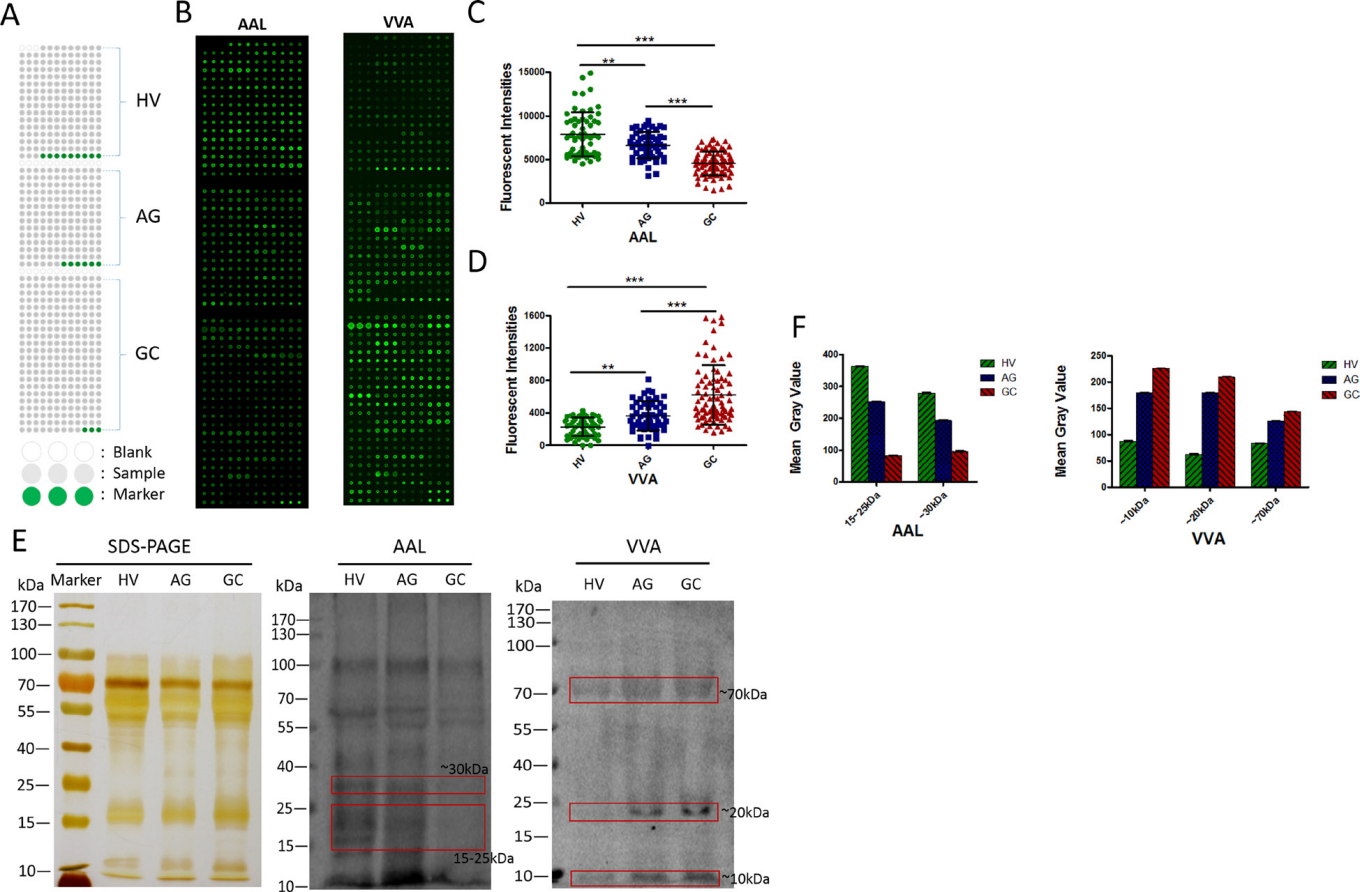
Validation of the differential expressions of the glycopatterns in the saliva among HV, AG and GC (**A**) The layout of a saliva microarrays. (**B**) Scan images of Cy5-labeled AAL and Cy5-labeled VVA bound to a saliva microarray, respectively. (**C**) The scatterplots of the original data obtained from the saliva microarrays incubated by Cy5-labeled AAL. (**D**) The scatterplots of the original data obtained from the saliva microarrays incubated by Cy5-labeled VVA. (**E**) Binding pattern of glycoproteins from salivary samples of HV, AG and GC using Cy5-labeled AAL and Cy5-labeled VVA. (**F**) The mean gray value of each apparent difference bands were read by ImageJ.

To rapidly validate the phenomenon of AAL and VVA that exhibited the significantly different glycopatterns in GC compared with HV and AG, the salivary microarrays were developed to detect the gastric cancer-associated glycopatterns in individual saliva samples. Totally, 201 individual samples including HV (*n* = 60), AG (*n* = 54), and GC (*n* = 87), nine blanks and six markers were spotted in a slide (Figure 3A). The results were shown in Figure 3, AAL staining showed decreased tendency among HV, AG and GC (Figure 3B and 3C), while VVA staining showed increased tendency (Figure 3B and 3D). These results were basically coincident with the results from the lectin microarrays.

To further confirm the results of the salivary microarrays, the lectin blotting analysis were performed with AAL and VVA staining, respectively. The results of SDS-PAGE demonstrated that the salivary protein bands from patients were similar, except for an apparent different band with molecular weight (Mr) of approximately 25 kDa as compared with the HV (Figure 3E). The AAL staining showed a decreased binding tendency from HV, and AG to GC subjects according to three apparent bands (red frames) ranging from 15 to 40 kDa, while the VVA staining showed a increased binding tendency from HV, and AG to GC subjects according to three apparent bands (red frames) with Mr of approximately 10 kDa, 20 kDa and 70 kDa (Figure 3E and 3F). These results were basically coincident with the results from the saliva microarrays and lectin microarrays.

### Construction of diagnostic models based on glycopattern abundances

The GC- and AG-related salivary glycopatterns were evaluated based on the above 15 candidate lectins that exhibited significantly alterations of salivary glycopatterns with the development of GC in test group.

The Model GC mathematic formula was constructed to differentiate the GC from the HV and AG using logistic regression analysis [25, 26].

$$\text{Model GC}=\frac{1}{1+e^{-(-7.866+233.473*\text{VVA}+342.447*\text{SBA})}}$$

**Figure 4:**
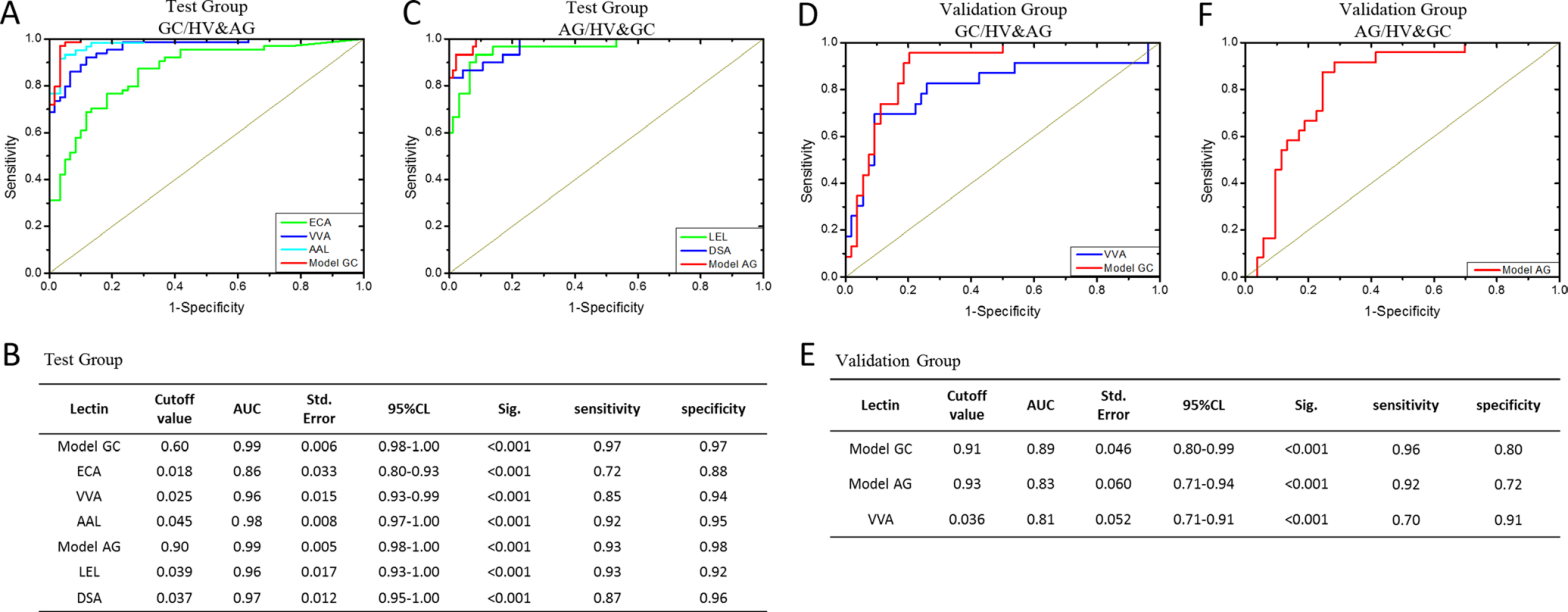
The diagnosis accuracy of the selected lectins and models analyzed by ROC analysis (**A**) The ROC analysis for the candidate lectins and Model GC in the test group. (**B**) The detail information of the ROC analysis for the constructive models and candidate lectins in the test group. (**C**) The ROC analysis for the candidate lectins and Model AG in the test group. (**D**) The ROC analysis for the candidate lectins and Model GC in the Validation group. (**E**) The detail information of the ROC analysis for the constructive models and VVA in the Validation group. (**F**) The ROC analysis for Model AG in the Validation group.

The diagnostic accuracy of Model GC referred to two lectins (VVA and SBA) and the selected lectins in the test group were analyzed by ROC analysis (Figure 4A and 4B). The ROC curves indicated that Model GC and three candidate lectins (ECA, VVA and ALL) had higher diagnostic accuracy for distinguishing GC from HV and AG, such as Model GC (AUC: 0.99, sensitivity: 0.97, and specificity: 0.97), ECA (AUC: 0.86, sensitivity: 0.72, and specificity: 0.88), VVA (AUC: 0.96, sensitivity: 0.85, and specificity: 0.94) and AAL (AUC: 0.98, sensitivity: 0.92, and specificity: 0.95). Notably, Model GC had the highest diagnostic accuracy for distinguishing GC from HV and AG.

The Model AG mathematic formula was constructed to differentiate the AG from the HV and GC using a logistic regression analysis.

$$\text{Model AG}=\frac{1}{1+e^{-(11.319-164.630*\text{DSA}-120.286*\text{LEL})}}$$

The diagnostic accuracy of Model AG referred to two lectins (DSA and LEL) the selected lectins in the test group were analyzed by ROC analysis. The ROC curves indicated that Model AG and two candidate lectins (LEL and DSA) had higher diagnostic accuracy for distinguishing AG from HV and GC (Figure 4C and 4B), such as Model AG (AUC: 0.99, sensitivity: 0.93 and specificity: 0.98), LEL (AUC: 0.96, sensitivity: 0.93, and specificity: 0.92) and DSA (AUC: 0.97, sensitivity: 0.87 and specificity: 0.86). Notably, Model AG had the highest diagnostic accuracy for distinguishing AG from HV and GC.

### Evaluation of the diagnostic models

The constructive models and selected lectins in the test group were then applied to the validation group of patients with GC (*n* = 23) and AG (*n* = 24), and of HV (*n* = 30) to evaluate the diagnostic power. ROC analysis were performed to show the diagnostic accuracy of the constructive models and candidate lectins. The ROC curves indicated that the Model GC (cutoff value: 0.91, AUC: 0.89, sensitivity: 0.96 and specificity: 0.80) had high diagnostic accuracy for distinguishing GC from HV and AG (Figure 4D and 4E). 22 cases of 23 GC and 19 cases of 24 AG as well as 24 case of 30 HV were correctly classified by Model GC. While the ROC curves indicated that the Model AG (cutoff value: 0.93, AUC: 0.83, sensitivity: 0.92 and specificity: 0.72) also had high diagnostic accuracy for distinguishing AG from HV and GC (Figure 4F and 4E). 22 cases of 24 AG and 13 cases of 23 GC as well as 25 case of 30 HV were correctly classified by Model AG. However, there was only VVA in all selected lectins, its ROC curve achieved a better diagnostic power (cutoff value: 0.036, AUC: 0.81, sensitivity: 0.70 and specificity: 0.91) with an AUC value greater than 0.80 for distinguishing GC from HV and AG (Figure 4D and 4E). 16 cases of 23 GC and 22 cases of 24 AG as well as 27 case of 30 HV were correctly classified by VVA.

## DISCUSSION

There is no doubt that glycans carry huge information, but our understanding of their functions has still lagged. Fortunately, more and more researchers turn their attention to the glycans, making a deep cooperation in interdisciplinary and propelling forward our understanding of glycans. Many reports indicated that the alteration of glycans affects the function of glycoproteins, for example, E-cadherin aberrant N-glycosylation at Asn-554 was demonstrated to affect its critical functions, and the aberrant glycans could be used as potential biomarkers [27, 28].

In our study, 124 saliva samples were collected for screenning candidate biomarkers and constructing diagnostic models in the test group. While other 77 saliva samples were collected for examining diagnostic accuracy of the selected candidates in the validation group. There were 15 lectins (e.g., PSA, PHA-E and ECA) that revealed significant alterations of the salivary glycopatterns among HV, AG and GC through statistical analysis. The results indicated that the expression level of fucosylation including outer-arm fucosylation and core-fucosylation recognized by AAL and PSA in saliva was down-regulated in GC compared with HV, which was coincident with many reports in serum and tissue of GC [6, 29]. Furthermore, the AAL-specific glycans decreased in GC were confirmed by saliva microarray and lectin blotting analysis.

In addition, the expression levels of Gal and GalNAc structures recognized by PNA, EEL, MPL, GSL-I, BS-I, ECA, SBA, and VVA in saliva was up-regulated in GC compared with HV. And we also confirmed that the VVA-specific glycans were increased in GC by saliva microarray and lectin blotting analysis. Similarly, Tn antigen and its derived structure T antigen also up-regulated in serum and tissue of GC [21, 30], which was associated with the invasion and metastasis of cancer [31]. Our results also showed that the expression levels of T and Tn antigen significantly increased in AG compared with HV, which positively correlated with the development of gastric disease.

Further, we constructed the diagnostic models of GC and AG using 15 selected lectins. ROC analysis was performed to show the diagnostic accuracy of the candidate lectins and the diagnostic models. Three lectins (ECA, VVA, AAL) and Model GC with AUC value greater than 0.80 (*p* < 0.001) were able to distinguish GC from HV and AG in the test group. While two lectins (LEL and DSA) and Model AG achieved a desired diagnosis power for AG from HV and GC in the test group. All selected lectins and the diagnostic models were further evaluated for the accuracy of the diagnosis of GC and AG in validation group. The Model GC and Model AG were more stable and reliable, and achieved better diagnostic power with an AUC value greater than 0.80 (*p* < 0.001) for the diagnosis of GC and AG than that of the single lectin in the validation group. We have also considered ratio as a metric for distinguishing these populations. For example, VVA binding glycoproteins were higher in cancer and lower in volunteers, while AAL binding glycoproteins were lower in cancer and higher in volunteers. VVA/AAL ratio might be a metric for distinguishing these populations. Likewise, DSA or LEL versus LTL might be useful for gastritis. The results showed that VVA/AAL, DSA/LTL and LEL/LTL can get a good performance in test group with an AUC value greater than 0.80. But they were not perfectly suited for validation group (Supplementary Figure 1).

Interestingly, the PCA result showed that there was a small overlapping area between AG and GC pools in our study (Figure 1D), indicating that the glycopattern expression levels of AG were partly similar to GC in the saliva, which might imply an early stage of a malignant transformation from AG to GC. Thus PCA was used to analyze the relationships just between AG and GC, which can differentiate almost all AG from GC cases, except two samples, based on salivary glycopatterns (Supplementary Figure 2). Patients with AG located in this overlapping area maybe have a very high canceration risk.

In conclusion, the present study investigated the alterations of salivary glycosylation related to the development of GC, and systematically compared different or similar alterations of salivary glycopatterns among HV, AG and GC, as well as the Model GC and Model AG with high diagnostic accuracy (Model GC (AUC: 0.89, sensitivity: 0.96 and specificity: 0.80), Model AG (AUC: 0.83, sensitivity: 0.92 and specificity: 0.72)) were constructed based on 15 selected lectins (e.g., PSA, PHA-E and ECA) that exhibited significantly alterations of protein glycopatterns in saliva. This study provides pivotal information to distinguish HV, AG, and GC based on precise alterations in salivary glycopatterns, which have great potential to be biomarkers for diagnosis of GC.

## MATERIALS AND METHODS

### Test group

The collection and use of human whole saliva for research presented here were approved by the Ethical Committee of Northwest University (Xi'an, China), First People's Hospital of Chenzhou (Chenzhou, China), and University of South China (Hengyang, China). Written informed consent was received from participants for the collection of their whole saliva. This study was conducted in accordance with the ethical guidelines of the Declaration of Helsinki.

In total, 94 patients with GC (*n* = 64) and AG (*n* = 30) were recruited between 2013 and 2014. The diagnoses for all the enrolled patients were histopathologically confirmed. In the control group, 30 age- and sex-matched HV were enrolled during the same time period. The above patients and HV were used in the test group to construct diagnostic models. A summary of the patient and HV clinical characteristics was provided in Table 1. GC patients who received preoperative radiotherapy, chemotherapy, chemoradiotherapy or curative were excluded. The collection of human whole saliva protocol was according to the protocol [19, 20].

**Table 1:** Clinical characteristics of healthy volunteers and patients with atrophic gastritis and gastric cancer

Characteristic	Test Group			Validation Group		
	HV	AG	GC	HV	AG	GC
n	30	30	64	30	24	23
Age, y, mean ± SD	52.7 ± 7.4	52.3 ± 9.0	56.0 ± 9.2	52.9 ± 9.4	52.5 ± 8.9	55.3 ± 10.1
Sex, male/female	20/10	21/9	43/21	20/10	16/8	16/7
H.P. infection (positive/negative)		22/8	47/17		18/6	17/6
Pathological (AJCC)^a^						
I			21			7
II			18			8
III			25			8
IV						
Tumor (T), n,						
T1			21			8
T2			9			2
T3			2			1
T4			32			12
Node (N), n,						
N0			30			10
N1			6			3
N2			16			4
N3			12			6
Metastasis (M), n,						
M0			64			23
M1			0			0

^a^AJCC, American Joint Committee on Cancer staging system (7th edition).

### Validation group

To evaluate the predictive value of the models established in the test group described above, an additional cohort (*n* = 77) of HV (*n* = 30) and patients with GC (*n* = 23) and AG (*n* = 24) were prospectively investigated from October 2015 to March 2016 at the same hospital. A summary of the patient and HV clinical characteristics was also provided in Table 1.

### Lectin microarrays

A lectin microarray was produced using 37 lectins with different binding preferences covering N- and O-linked glycans [19]. The Cy3-labeled proteins were incubated in lectin microarray to detect the different glycopattern among clinical samples. The lectin microarrays were produced according to the protocol [19].

### Saliva microarrays

A saliva microarray was produced by 201 individual saliva samples including HV (*n* = 60), AG (*n* = 54), GC (*n* = 87) according to the protocol [19]. The Cy5-labeled lectins (VVA and AAL) were applied to detect the specific glycan structures in the saliva samples which immobilized on the slides.

### Lectin blotting

The expression levels of glycan structures were analyzed by lectin blotting according to the protocol [19]. The pooled salivary proteins of each group were subjected to 10% SDS-PAGE electrophoresis, and transferred to PVDF membranes incubated with the Fucα1-6GlcNAc(core fucose) and Fucα1-3(Galβ1-4)GlcNAc binder AAL and the terminal GalNAc, GalNAcαSer/Thr(Tn) and GalNAcα1-3Gal binder VVA, respectively.

### Statistical analysis

The original data of lectin microarrays need to be normalized for minimizing the possible systematic variation. The background was subtracted, and values less than the background ±2 standard deviations (SD) were removed from each data point. The median of the effective data points for each lectin was globally normalized to the sum of medians of all effective data points for each lectin in a block, which were named normalized fluorescent intensities (NFIs).

Statistical differences between groups were first assessed using a Kruskal-Wallis test, followed by a Dunn's Multiple Comparison Test to correct for multiple comparisons using GraphPad Prism5.0 software. Differences were considered statistically significant for values of *P < 0.05, **P < 0.01 or ***P ≤ 0.001. Spearman's correlation coefficients was performed to evaluate the correlations between lectins and gastric disease using SPSS statistics 21.0 software. Following the normalized data was further analyzed by unsupervised average hierarchical cluster analysis (HCA) using Expander 6.0 (http://acgt.cs.tau.ac.il/expander/) and principal component analysis (PCA) using Multi-Variate Statistical Package (UK). Model GC and Model AG were constructed according to the glycopattern abundances based on a forward stepwise logistic regression analysis using SPSS statistics 21.0 software. The diagnostic performance of candidate lectins and diagnostic models was evaluated by ROC curve analysis using Origin 8.0 software.

## SUPPLEMENTARY MATERIALS FIGURES



## Abbreviations

GC: Gastric Cancer
AG: Atrophic Gastritis
HV: Healthy Volunteers
Model GC: Diagnostic Model of GC
Model AG: Diagnostic Model of AG
NFIs: Normalized Fluorescent Intensities
SD: Standard Deviations
HCA: Hierarchical Cluster Analysis
PCA: Principal Component Analysis
ROC: Receiver Operating Characteristics
AUC: Area Under the Curve
