# Supplementary material for: Salivary glycopatterns as potential biomarkers for diagnosis of gastric cancer
Source: Oncotarget. 2017 Mar 10;8(22):35718–27. doi: 10.18632/oncotarget.16082 (PMC5482611; doi:10.18632/oncotarget.16082)
Supplement: Supplementary file 1 [file oncotarget-08-35718-s001.pdf]

Salivary glycopatterns as potential biomarkers for diagnosis of gastric cancer

Supplementary Materials

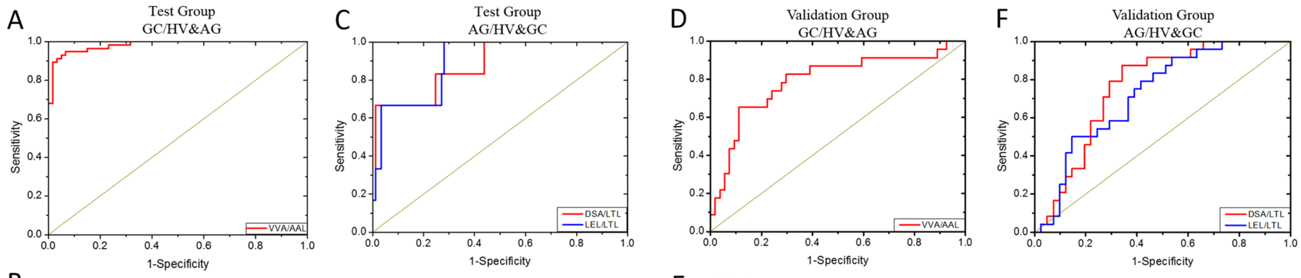

B Test Group

	AUC	Std. Error	95%CL	Sig.	Specificity	Sensitivity
VVA/AAL	0.98	0.010	0.96-1.00	<0.001	0.93	0.95
DSA/TLT	0.88	0.068	0.75-1.00	0.0019	0.99	0.67
LEL/LTL	0.89	0.076	0.75-1.00	0.0013	0.71	1.00

E Validation Group

	AUC	Std. Error	95%CL	Sig.	Specificity	Sensitivity
VVA/AAL	0.79	0.056	0.68-0.90	<0.001	0.89	0.65
DSA/TLT	0.76	0.069	0.63-0.90	<0.001	0.66	0.88
LEL/LTL	0.73	0.070	0.59-0.86	0.0026	0.61	0.75

Supplementary Figure 1: The diagnosis accuracy of the ratio stratetgy analyzed by ROC analysis. (**A**) The ROC analysis of VVA/AAL in the test group. (**B**) The detail information of the ROC analysis for the in the test group. (**C**) The ROC analysis of DSA/LTL and LEL/LTL in the test group. (**D**) The ROC analysis of VVA/AAL in the Validation group. (**E**) The detail information of the ROC analysis in the Validation group. (**F**) The ROC analysis of DSA/LTL and LEL/LTL in the Validation group.

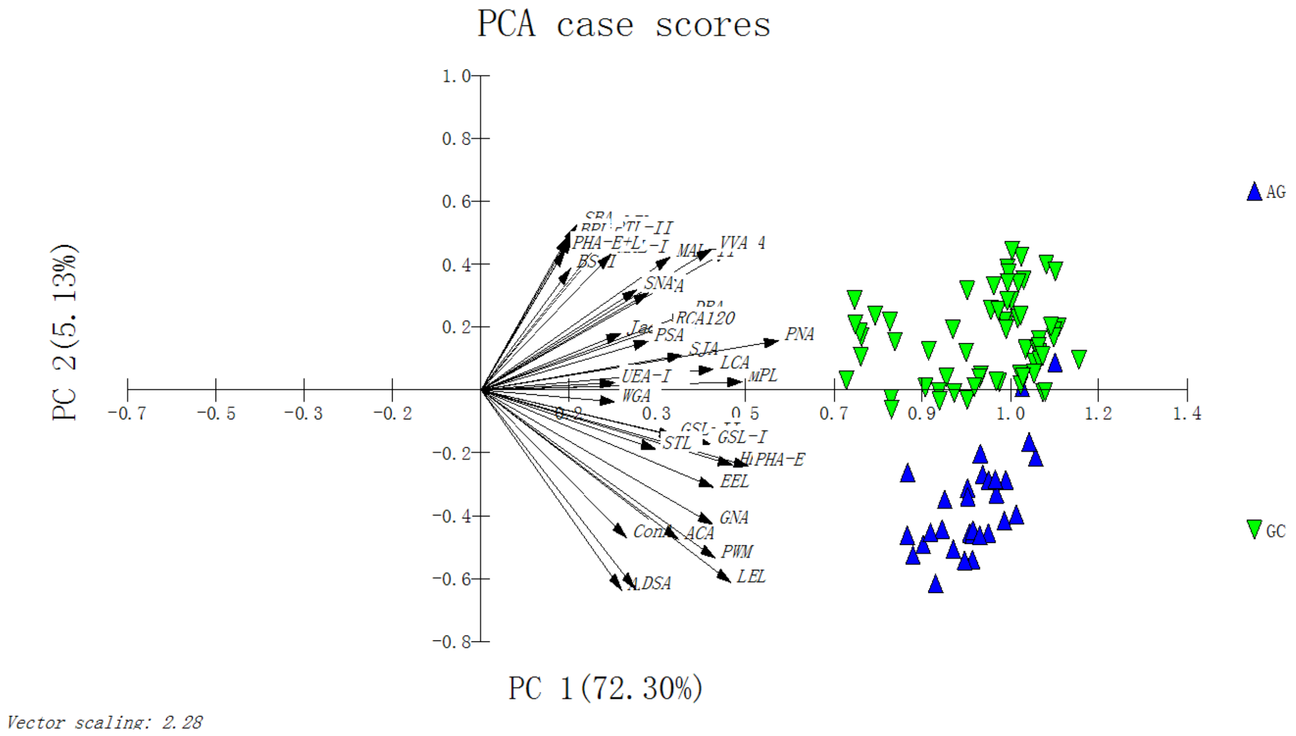

Supplementary Figure 2: The result of PCA analysis between AG and GC.
